# Around the Hospitals

**Published:** 1894-10-13

**Authors:** 


### Around the Hospitals.

[Notice to the Managers of Institutions.—Special space is now reserved for the insertion of notices of hospital meetings and festivals. The Editor requests that all notices may reach The Hospital Office, 428, Strand, at least one week before the date of each meeting.]

**Guy's Hospital.**—The report from Guy's Hospital is most interesting reading. It fully shows the intelligence and deep interest with which it has been compiled, and which are evidently at work also throughout Dr. Perry's administration of the hospital. It is a well known fact that funds are very restricted at Guy's, but ingenuity and care have supplemented the limited sums at command, and many necessary improvements have been effected at small cost. By judicious co-operation with the St. Olave's Board of Works an excellent Washington-Lyon Disinfector has been secured to serve for the Board and the hospital, and thus minimise the expense of the necessary and thorough disinfection of clothes, bedding, and other articles. Another necessary appliance has been secured in an equally economical manner. A very satisfactory destructor furnace has been devised, which, whilst disposing of all refuse, at the same time acts as a generator of steam to drive the engine required to pump the water from an artesian well, and also to work the machinery in the laundry. The contrivance was erected at a cost of £100, and has been the means of saving thirty tons of coal during six months. This fact might well be noted by the managers of hospitals generally. The hospital now prepares its own aërated waters in the dispensary. About £30 per annum was expended under the old system, whilst the new method appears to work out at about £20. Whilst speaking of improvements we must not omit to mention the system which now connects many of the wards with the house surgeon's residence in the college, by means of speaking-tubes and telephones, a most valuable and necessary arrangement in connection with hospital work. Great pains have been taken to render the nursing department as efficient and satisfactory to the nurses as possible, Miss Nott Bower having proved the wisdom of her appointment by the excellence of the services she has already rendered as Matron. Several changes have taken place in the medical staff and elsewhere in the hospital. Mr. Arthur E. Durham retiring, has been succeeded by Mr. Golding-Bird as surgeon to the hospital, the vacant post caused by this promotion being filled by Mr. L. A. Dunn, now assistant surgeon. The number of beds available is 518, two wards being unoccupied for want of funds. The number of cases treated within the hospital during the year shows an increase, and amounted to 6,160. In the out-patients' department the growth is still more visible, and shows that the 23,550 attending during 1893 exceeded those of the previous year by upwards of 1,700. The increase in numbers does not show a corresponding improvement in the income of the hospital. The rents from landed property fell to the lowest point yet reached, amounting to little more than half the sum received fourteen years ago. The deficiency on the year's expenditure amounting to £8,473, was made up from the special appeal fund. The income was £27,788, the expenditure £36,630. The average cost per occupied bed amounted to £82, the average cost per in-patient was £5 10s. 1d.

